# Parents’ multi-layered expectations when requesting an Autism Spectrum Disorder assessment of their young child: an in-depth interview study

**DOI:** 10.1186/s12888-020-02806-7

**Published:** 2020-09-10

**Authors:** Delphine Jacobs, Jean Steyaert, Kris Dierickx, Kristien Hens

**Affiliations:** 1 Faculty of Medicine, Centre for Biomedical Ethics and Law – KU Leuven, Kapucijnenvoer 35 box 7001, 3000 Leuven, Belgium; 2 Centre for Autism Expertise, Child and Youth Psychiatry – University Hospitals Leuven, Herestraat 49, 3000 Leuven, Belgium; 3 Department of Philosophy, Faculty of Arts, University of Antwerp, Prinsstraat 13, 2000 Antwerpen, Belgium

**Keywords:** Autism spectrum disorder diagnosis, Parents, Interviews, Young child, Experiences, Expectations

## Abstract

**Background:**

Parents are valued stakeholders in research, clinical practice and policy development concerning autism spectrum disorder (ASD). However, little is known about what drives and moves parents besides their obvious worries and help request when they ask for a diagnostic ASD assessment of their child.

**Methods:**

Seventeen Flemish parents of 11 young children participated in a longitudinal study consisting of three in-depth interviews before and after their child’s diagnostic ASD assessment. Data were analysed in Nvivo 11 according to the procedures of Interpretative Phenomenological Analysis.

**Results:**

In this paper we report the results of the first series of interviews which were conducted after parents had asked for an ASD assessment of their young child, and before this assessment started. The pre-assessment experiences of the parents were dominated by the anticipation of various implications of an ASD diagnosis, comprising both positive and negative expectations. The theme of positive expectations consisted of two equally prominent subthemes: treatment-related implications but also expectations pertaining to their psychological and relational experiences.

**Conclusions:**

This study suggests important issues for clinicians to bear in mind during a consultation with parents who request an ASD assessment of their young child. We argue that attending to and communicating about parents’ expectations prior to their child’s ASD assessment may help clinicians to better understand parents’ requests for help, and to address their needs more effectively.

## Background

Autism Spectrum Disorder (ASD) is defined as a neurodevelopmental condition characterised by early-onset difficulties in social communication and by repetitive behaviours and restricted interests [1]. Philosophical and historical research has shown that ASD’s definition is not unequivocal and has taken different forms over time [2, 3]. Also, its validity has been called into question, as there is no certainty as to whether autism actually refers to an underlying delineated biological reality [4–7]. ASD has therefore been called a ‘problematic category’ [8] and ‘a contested illness’ [9]. Nevertheless, ASD’s biological -genetic and neurological- reality is *not* under question, regardless of whether ASD is considered to represent either a disorder (as in the Diagnostic and Statistical Manual of mental disorders) or a difference (as in the neurodiversity movement) [1, 10, 11]. Indeed, in most of Western psychiatry and medicine, ASD is quite uniformly understood as a biomedical condition which is diagnosed clinically in a multidisciplinary assessment assisted by specific diagnostic tests [12].

In the last four decades there has been a significant rise in the prevalence of ASD diagnoses. The current prevalence is estimated to be at least 1.5% in developed countries [13, 14], and this prevalence led to a surge in investment in autism research [15]. However, after three quarters of a century of research and clinical experience, scientific and clinical findings continue to present ASD as a complex and heterogeneous condition in its presentation, cause and cognitive mechanism, treatment, and prognosis [11, 16–19].

When their child gets an ASD diagnosis, parents might want their clinician to tell what ASD is, what its cause and prognosis are, and what treatment is indicated. But a clinician cannot resort to unequivocal research findings and evidence-based clinical guidelines when answering these questions [20–23]. Most importantly, it is unclear in what way the diverse research findings, clinicians’ communications, and other influences concerning ASD (coming from the parents’ social network, child professionals in healthcare and education, and -social- media) are translated into information that is useful and meaningful for parents in their child-rearing practices and relationship with their child. In this regard, it has been stated that understanding ASD ought to begin with listening to, communicating with, and learning from ‘autistic’ people and their parents, and by understanding their experiences [24, 25]. Indeed, several authors have warned against the growing gap between basic ASD science on the one hand, and clinical practice and the community on the other [17, 26]. We argue that it is therefore necessary to include the voice of the patients -their parents in the case of very young children- in the debate on good clinical care -even more so than in medical fields with clear and uniform research results.

Several studies have already investigated parents’ experiences of having a child with an ASD diagnosis. They investigated for example the stress, stigma and burden experienced by parents when caring for a child with ASD [27, 28] or they focused on parents’ satisfaction with the diagnostic process [29, 30]. Empirical studies rarely investigated how parents understand an ASD diagnosis and experience the process of their child getting an ASD diagnosis. Historically, a medical and a social model of disability is distinguished in concerning the understanding of disabilities [31, 32], and in Anglo-Saxon countries, the neuro-diversity movement is an influential voice in the debate on how to understand ASD [33]. In Flanders however, this debate on how to understand disabilities or ASD is not publicly conducted nor was any previous research done on this topic in Flanders. A small number of empirical studies, mostly conducted in the US and the UK, did investigate this topic [31, 34]. In reviewing these latter studies, we found a temporal shift in parents’ understanding and experiences of ASD throughout their trajectory in ASD care; and found that parents might expect several implications of an ASD diagnosis that pertain to the psychological and relational domain [35–38]. These findings inspired the research question and design of this study.

Thus, the aim of this study is to explore and gain an insight into how parents of young children understand and experience an ASD diagnosis during the waiting period prior to their child’s diagnostic ASD assessment. Because our review showed a temporal evolution in parents’ understanding and experiences of ASD, we set up a longitudinal study on parents’ experiences before and after the ASD diagnostic assessment of their child, by conducting 3 interviews: (T1) before the diagnostic assessment; (T2) right after the feedback session at the end of the assessment; and (T3) 12 months later. In this manuscript, we present the first part of this longitudinal study concerning the pre-assessment interviews. The longitudinal evolution from T1 to T2 to T3 is described in another article [39] which addresses the understanding and experiences of ASD throughout time by the included Flemish parents. This longitudinal study in turn is part of a broader research project which triangulates this study’s findings by investigating doctors’ understanding and clinical experiences of an ASD diagnosis [40–43].

## Methods

We conducted a longitudinal study consisting of three in-depth interviews with parents before and after their child’s diagnostic ASD assessment. In the current paper we report the results of the first series of interviews which were conducted after parents had asked for an ASD assessment of their young child, and before this assessment started.

This method section is similar to the method section in the article concerning the longitudinal evolution in parents’ experiences throughout three interviews [39].

### Sample

We included 17 Flemish parents of 11 children who requested a diagnostic ASD assessment of their child up to 6 years of age without a previously diagnosed disability (intellectual disability (ID) or other disability). In the Flemish Region of the federal state Belgium, health insurance is mandatory and covers the majority of all medically indicated procedures, like a diagnostic ASD assessment. Children need a DSM diagnosis in order to be entitled to specialised support and treatment. Half of toddlers attend day care, mostly state-funded, and from 2.5 to 3 years onwards, more than 90% of children go to a state-funded school. Until data saturation was achieved, and no new themes showed up, we purposively sampled participants [44–46]. The inclusion criteria consisted of being the parent of a child of 6 years or younger who asked for a diagnostic ASD assessment of his/her child in two specialist centres in Leuven: a child psychiatric and a paediatric diagnostic centre. The only exclusion criterion was that the child was not already diagnosed with a disability, intellectual or other. Parents are either referred to such a centre by a child professional or they self-refer. One of the clinicians who would be conducting the child’s assessment called the parents to invite both father and mother to participate in this study. Fifty percent of the invited parents, the parents of 11 out of 22 children, chose not to participate. In accordance with the study protocol approved by the UZ Leuven Ethics Committee, parents had the right to abstain from participating in the study, both before and in the course of the study, without giving an explanation. Information about the study was given orally and in written form, and all participants gave their informed consent for inclusion in the study and for publication of the study results. The Ethics Committee of the University Hospitals Leuven approved this study on 3 February 2017 (Belgian Registration Number B322201731147). This study was performed in accordance with the ethical standards laid down in the 1975 Declaration of Helsinki and its later amendments.

### Data collection

Data were gathered by conducting in-depth phenomenological interviews of one parent or of both parents together of a child. The interviews were semi-structured by a topic guide (Table 1) which we developed based on the findings of our literature review on how parents understand and experience ASD [37, 47, 48].

**Table 1:** Interview guide

Research questions:	
How do parents understand and experience a possible ASD diagnosis of their child?	
What do parents think and feel when they ask for a diagnostic ASD assessment of their child? During this interview, we are going to talk about possibly receiving an ASD diagnosis for your child. We would like to understand what thoughts and feelings you experience personally prior to your child’s diagnostic ASD assessment.	
Do you have any questions or comments regarding the information and consent forms?	
1. Can you describe how you view an ASD diagnosis? What does an ASD diagnosis mean to you?	
- Thoughts/feelings, before/during application phone call	
- Worries, child-rearing experiences, parent-child relational experiences	
- What request for help	
- What expectations	
2. Can you describe what ‘receiving an ASD diagnosis’ would mean to you and your child?	
- How do you understand the idea of your child getting an ASD diagnosis?	
- What do you feel to be the (wanted and less wanted) implications of a possible ASD diagnosis?	
- Impact of a possible ASD diagnosis on your life and that of your child	
° Would your life be different?	
° Would your child’s life be different?	
° What exactly would change?	
- Impact of a possible ASD diagnosis on how you view your child	
° How do you view your child, how do you talk to and interact with your child?	
° The child’s view of himself (i.e. later in life)	
° Would anything change with an ASD diagnosis, and what exactly?	
Are there things that are important to you which have not yet been addressed or things that I have forgotten to ask? Would you like to add something or change an answer?	

That is, the topic guide mainly concerned the parents’ -possibly evolving- understanding of an ASD diagnosis, and their expectations regarding the implications of their child’s ASD diagnosis with regard to treatment but also to their psychological and relational experiences. The interview guide was pilot tested, and consisted of open-ended questions. It was used flexibly, in order to encourage participants to elaborate freely and reflectively on a theme, either mentioned by the interviewer or a closely related one they wanted to talk about. The interviews were conducted in Dutch by the first author (DJ); she is a female child and adolescent psychiatrist (M.D.) who has been working clinically for over 15 years with this study’s population and has a special interest in the ethical issues of child and adolescent psychiatric diagnoses. No parent asked questions about the interviewer’s occupational background. Most interviews took place at the participants’ homes, two participants preferred to be interviewed in the Centre where they had been recruited. In 5 instances, the child the parents were talking about was partly present in the room during an interview. The interviews were audio recorded and lasted for 45 to 102 min, depending on the parents’ elaborations on the topic. Data were collected between March 2017 and January 2018.

### Analysis

We inductively analysed the data by applying the procedures outlined for IPA, a qualitative research method based on phenomenology, ideography and hermeneutics, and aimed at understanding the experiences and sense making of people around a life event [48] -in this case the ‘life event’ of having a child being diagnostically assessed for ASD. DJ made field notes right after the interviews and transcribed each recorded interview verbatim. DJ and KH systematically and inductively coded the transcripts line-by-line in NVivo 11 [49], identifying passages of text across the data of one interview that were related to the research question. Then, DJ and KH made a comparison of the relevant codes across all interviews in order to discern patterns and contradictions. Consequently, these primary codes were clustered into recurrent subthemes and synthesised into themes. All four authors met regularly to discuss and govern this analytical process in an interdisciplinary collaboration and specifically to decide what themes and subthemes were most prominent in the data. Finally, an overall narrative account was created by combining the dominant themes extracted from the interviews. We translated representative quotations verbatim from Dutch to English, in order to give an impression of the interviewees’ original expressions. Square brackets indicate information added from the interview material before or after the quote in order to facilitate readers’ understanding of the quote. Several strategies were employed to strengthen the validity and trustworthiness of the findings. For example, the analytic process was subjected to peer scrutiny by researchers from the local psychology and pedagogy faculties during several meetings and seminars; the data were triangulated by also interviewing physicians working with the same group of children -and their parents- with a similar research question; and by conducting 3 interviews with the same participant the study’s credibility was increased because of the improved potential to generate data which fitted the views of the participants and properly represented the dynamic process [40, 41, 50, 51]. The manuscript was subjected to the COREQ checklist for consolidated criteria for reporting qualitative research [52] (Additional file 1).

## Results

We interviewed 17 Flemish parents of 11 children between 1 and 6 years of age after the parents applied for a diagnostic ASD assessment of the child (Table 2).

**Table 2:** Sample characteristics

Child	Interviewed parent	Sibling with ASD diagnosis	Family structure
1	Mother	/ (first child)	Mother+Father
2	Mother+Father	+	Mother+Father
3	Mother+Father	/	Mother+Father
4	Mother+Father	/ (first child)	Mother+Father
5	Mother	/	Parents divorced, Father absent
6	Mother+Father	+	Mother+Father
7	Mother+Father	+	Mother+Father
8	Mother	+	Mother+Father
9	Mother	+	Mother+Father
10	Mother+Father	/	Mother+Father
11	Mother	+	Mother+Father
	Mother+Father	Sibling with ASD diagnosis	Mother+Father
	6/11	6/11	10/11
	(55%)	(55%)	(91%)

All but one children had been referred for a diagnostic assessment by a professional, and all but one parent couples agreed with the referring professional that a diagnostic assessment of their child was indicated. The average age of the 11 children at the moment of the first of three interviews was 3y08m, sd 1y05m. The parents were all native Flemish people, and their education and employment background were diverse. All children were male. We did not ask for or score the severity of their symptoms -leading to the request for an ASD assessment. As a matter of fact, to our knowledge, the severity score of the ASD diagnosis as added to the DSM-5 diagnostic criteria, is never scored in Flemish clinical practices. We only included children without a previous diagnosis of disability, intellectual or other. Six out of 11 children were found to have an older sibling who already had an ASD diagnosis: their parents are called ‘Sib+’ as opposed to ‘Sib-‘ parents who do not have an older child diagnosed with ASD. When we could interview both parents of a child (in 6 out of 11 cases), most fathers said that they were less concerned about their child’s development and less in demand of a diagnostic assessment. However, later in the interviews, fathers’ worries appeared to be quite similar to the mothers’ worries but fathers were clearly less preoccupied with other people’s critical remarks about the child and about themselves as parents (see below).

The pre-assessment experiences of parents followed a sequential course of, as a first theme, parents and professionals noticing worrisome behaviours in the child; second, parents involving professionals as well as, in parallel, professionals addressing parents; third, ASD getting mentioned and a diagnostic ASD assessment being arranged; and fourth, parents having ambiguous feelings about the (consequences of the) possible ASD diagnosis. In this paper, we will describe the latter theme in detail (the 3 other themes are described in [39]), consisting of anticipated positive and negative implications of the diagnosis. The positive part of this theme consisted of two equally prominent subthemes: treatment-related and ‘psycho-relational’ expectations (Additional file 2).

### Ambiguous feeling about (the consequences of) the diagnosis

The planned diagnostic assessment of the child and his possible ASD diagnosis often aroused ambiguous feelings in the parents we interviewed: parents expected the ASD diagnosis to have both negative and positive implications.

### Anticipated negative implications of an ASD diagnosis

The expected negative implications pertained to two categories: (1) understanding ASD as being a condition ‘for life’; and (2) fearing social reactions of stigmatising and stereotyping the child.

First, both Sib + and Sib- parents were afraid that their child would get an ASD diagnosis because this would entail the attribution of a ‘life-long’ biological condition, ‘inside there’.

*Yes, it is, yes, for life, you know, just like you have your personality for life, you know. You can work on it but you will never be able to take it away entirely, it will stay inside there, you know.* –Mother 3

However, some parents said that they preferred an ASD diagnosis over a diagnosis of ID because of their perception of ID being more ‘fixed’ than ASD, less amenable to progress, like the following mother said:

*I don’t know, in the case of mental retardation [ID] anyway yes, then it is fixed, the child won’t catch up, he will always have it. But in the case of autism, yes, for me it’s more like, look, if we know it, we can act accordingly.* –Mother 10

Second, parents feared other people’s stereotypical ideas about a diagnosed child, for example seeing all of the child’s behaviours as due to ASD, and feared the social reactions of people stigmatising both them and the child. The following mother explained her fears in this regard concerning her child who was going to be assessed. She had 3 children. The oldest one was diagnosed with a developmental syndrome with a genetic cause and a visible facial difference. He was attending a special needs school.

*I’m afraid of [Child going to have a diagnostic ASD assessment] getting ‘a stamp’ at school, and that maybe [the teachers] will deprive him of possibilities. Some people will only see his autism and not the rest of him anymore, and act accordingly, even if he develops unexpectedly well.* –Mother 10 (Sib-)

The fear of stigma and the fear of stereotyping were present both in Sib- parents and Sib + parents, but for Sib + parents this anticipation was validated by previous experiences with the older child. The next mother had several older children with an ASD diagnosis. She -and the child’s father- talked at length about their experiences with the older children:

*We experienced that some [siblings with an ASD diagnosis] got ‘put in a box’. Everything that goes wrong: ‘Yes, but he’s autistic.’ And then I think, ‘But not everything has to do with autism’. You have to look at it more broadly. Sometimes the teacher can make an effort as well, even if it is autism, you know.* –Mother 2 (Sib+)

### Anticipated positive implications of an ASD diagnosis

Parents anticipated several positive consequences of an ASD diagnosis. Sib + parents and Sib- parents anticipated very similar implications, but again, for Sib + parents this anticipation was validated by previous experiences with their older child with an ASD diagnosis. The anticipated positive consequences consisted of treatment-related and psycho-relational implications.

#### Anticipated treatment-related implications of an ASD diagnosis

The parents we interviewed overall anticipated two potential implications with regard to how the child would be treated once diagnosed: (1) they were all very much aware that an ASD diagnosis entitled their child (and them) to receive services, at home, at school and in specialised treatment and guidance centres; and (2) they expected to receive professional advice concerning child-rearing practices that were more appropriate for the child. Especially Sib- parents wanted to adjust their parenting techniques at home, and both Sib- and Sib + parents very much wished for suited adaptations at school. For all Sib + parents, the predominant positive previous experience with their older child indeed had been that teachers were more willing to adapt their interaction with the child according to his needs once he had been diagnosed with ASD, because this diagnosis implied an objectivation of the child’s needs. The following quotes illustrate both treatment-related expectations:

*But it would make things easier, really, especially because we can knock at some door and say: we have that paper, we are really entitled to receive help. Besides that, [an ASD diagnosis] wouldn’t make any difference. You have to threaten the school with the label.* –Mother 9 (Sib+)

*We said, ‘We will proceed with this’ and then maybe we will find ways to handle him and make him obey in a pleasant way, without it turning into a battle each time. In the end you have tried every single approach, we have tried so much, received so much advice.* –Mother 4 (Sib-)

#### Anticipated ‘psycho-relational’ implications of an ASD diagnosis

Both Sib + and Sib- parents also anticipated many ‘psycho-relational’ implications of an ASD diagnosis, related to their psychological and relational experiences. The most frequently mentioned implications were: (1) understanding; (2) recognition; (3) reduced expectations -towards themselves as parents and towards the child; and (4) exculpation -of themselves and of the child.

First, many parents expected to better understand the behaviours of their child once he would be diagnosed with ASD, in two ways: having an explanation of the child’s behaviours, but also being more tolerant towards them.

*Maybe our vision of him will change, because maybe [with a diagnosis] we will understand him better, how he experiences things and actually receives things. That this is different after all. And how we can adapt our approach accordingly.* –Father 4

Moreover, these parents felt that such an understanding was really necessary for people around them. Also, two mothers thought this understanding would contribute to the child’s better self-understanding or self-knowledge once he would be older, like the following mother with a teenage daughter with an ASD diagnosis:

*Yes, because these are two people [the child and his older sister with an ASD diagnosis] who always think: it’s the other person [‘s fault]. And if you know this about yourself [that you have ASD, then you will think:] ‘I also have a share in this [conflict] although I am not always aware of it. But maybe I have to ask the other person: am I doing something wrong or am I not adapting enough to you?’* –Mother 8

When parents elaborated on ASD’s causational explanation, they always understood it as a mainly heritable, but certainly biological -genetic and/or neurological- condition.

*For me, yes, I can describe it very concisely: strange behaviour.* –Father 10

*And strange behaviour as a consequence of…?* –DJ

*A kink in the brain that’s not right. That’s my simple argumentation.* –Father 10

Second, several parents felt that an ASD diagnosis would provide some social recognition of their concerns about the child’s behaviours by objectifying these concerns. They expected that the recognition of their concerns would be soothing to themselves, and would also have a positive effect on the opinions and reactions of other people because they would view the parental concerns as legitimate. The following mother and her only child had a difficult time in the day care centre. The child carers told her that her son was not sleeping and eating well in the centre because he was stubborn.

*I kind of have, um, I want to know something, so not just being told ‘Yes, he is indeed a stubborn one’. Um, I really want to know. In that case [when he will be diagnosed with ASD], a burden will really be taken off my shoulders.* –Mother 1

Third, a diagnosis was found to regularly make parents temper their expectations both towards their own child-rearing practices, and towards their child’s good behaviours and development. In other words, the parents expected not to think anymore that their child-rearing practices had to meet the same standards (i.e. as before, and as with ‘normal’ siblings and other children). Moreover, the child would not be required anymore to meet the same expectations as other children of his age, both on behalf of the parents and of other adults (like teachers and family members). Often parents, both Sib + and Sib-, had already been adapting their educational practices towards the child (i.e. before the diagnostic assessment started), but a diagnosis was experienced as a proof that the parents were right to do so, for example by affirming that they were not spoiling the child by reducing their expectations. The following mother had 2 children. Her 3-year old daughter already had an ASD diagnosis. She told how she adapted her educational “principles”:

*I can still be angry, you know, for sure (laughing). But I learn a lot from [the child and his sibling with an ASD diagnosis], I learn to downplay many things, I don’t make a struggle out of everything, as I would have done before. Before, I would have stuck to my principles. Now, I am adapting my former ideal image about education. (laughing)* –Mother 11

Fourth, parents often expected a diagnosis to lift blame both from themselves and from their child. A diagnosis might absolve the parents and their child-rearing practices from responsibility for the difficult or different behaviours of the child. Also, these parents felt that their child would no longer be accountable for these behaviours if he would be given an ASD diagnosis. To the following mother (already cited above) the child carers from the day care centre said that her son was annoying and spoiled i.a. because he was allowed to sleep in the parents’ bed:

*Suppose it really is autism or something like that, then I will send a fierce email [to the day care centre] to tell that ‘my stubborn, annoying child who gets spoiled by me’ has a* condition*.* –Mother 1

## Discussion

This study showed a sequence of experiences of the interviewed parents prior to the diagnostic ASD assessment they requested for their young child. Especially the various implications the parents expected of the child’s possible ASD diagnosis stood out. Before discussing this main theme, we will shortly discuss the high proportion of participants having an older child diagnosed with ASD.

### Older sibling with an ASD diagnosis

Of the 11 children whose diagnostic process the parents were interviewed about, six were found to have an older sibling who already got an ASD diagnosis. Research has shown that ASD is more frequent in siblings of a child with ASD, with recurrence estimates up to 18.7% [53]. Moreover, not-related children living very close to a child previously diagnosed with ASD appear to be more likely to be diagnosed with ASD: a ‘social influence mechanism’ has been calculated to contribute to 16% of the increase in ASD prevalence over 2000–2005 in California [54]. The authors attribute this proximity effect to information diffusion about ASD. However, many symptoms that are common to ASD are not specific to ASD, and have been found to occur in children with other disorders [55]. So, the child’s behaviours leading the parent participants to ask for a diagnostic ASD assessment, might turn out to pertain to another diagnosis.

We found that having an older child with an ASD diagnosis influenced the interviewed parents’ understanding and experiences of an ASD diagnosis, but not predominantly at theme or subtheme level (Additional file 2). For example, Sib + parents had the same positive and negative expectations of an ASD diagnosis as Sib- parents, but these expectations had often been validated because the parents had already experienced several implications of the diagnosis with regard to their older child with an ASD diagnosis.

### Ambiguous feelings about the (consequences of the) diagnosis

The interviewed parents expressed ambiguous feelings once they had arranged a diagnostic ASD assessment for their child. They anticipated both positive and negative implications of the diagnosis if their child would be diagnosed with ASD.

On the negative side, several parents feared an ASD diagnosis of their child because of their understanding of ASD as a ‘life-long’ condition. This for-life account of ASD has been nuanced by recent research. For example in a community sample, one third of adults who was diagnosed with ASD (and not with ID) as children no longer had obvious ASD features [56, 57]. Such findings might be considered to advocate a more dynamic concept of ASD e.g. depending on an individual’s actual dysfunctioning at a given moment, like required in the DSM criteria [1, 58].

Parents also feared social reactions of stigmatising and stereotyping. Indeed, biomedical and neuroscientific conceptualisations of psychiatric diagnoses have been found to be associated with both increased and decreased stigmatisation [59–61]. Parents talked about the stigma of their child but also about the stigma towards themselves. In this regard, it has been suggested that a ‘broader phenotype’ and ‘assortative mating’ in adults with autistic-like characteristics might contribute to the ASD diagnosis of these adults’ children [62]. Such a suggestion might contribute to parents fearing to be stigmatised because of their child’s ASD diagnosis.

With regard to stereotyping, some parents feared that their child would be reduced to being ‘autistic’ and that, for this reason, teachers would expect less of them. Indeed, people whose disorders are characterised mainly by neuro-cognitive deficits have been found to be stereotyped as incompetent [63].

On the positive side, along with their fears concerning their child’s ASD diagnosis, all interviewed parents mainly expected to experience several positive implications of an ASD diagnosis. We discuss the four most frequently anticipated psycho-relational implications.

First, many parents expected an ASD diagnosis to make them and others *understand* their child’s behaviours. Two mothers also mentioned the explanatory capacity of a diagnosis for the child when he is older, i.e. the older child’s self-understanding might be better. An ASD diagnosis does not carry a real explanatory capacity in the sense that the underlying etiologic cause of the child’s behaviours and their psychopathological pathway usually becomes clear [5, 57]. This is not what parents meant when they talked about ‘understanding’. Instead, these parents seemed to say that [1] some biological condition would be determining the child’s behaviours and could thus explain his behaviours, and that [2] the diagnosis of ASD might make them and others more tolerant in their interactions with the child. Indeed, our findings are in line with those of other authors who reported that parents do not worry too much about the scientific explanation of ASD. Rather, “etiologic conceptions are mobilised according to specific needs” ([11], p95), like parents having the need to understand the child’s behaviours as determined by a biological condition. However, Hyman warns against an unwarranted reification of DSM diagnoses, by which a description of special behaviours by DSM criteria gets to be interpreted as representing a real and natural ‘thing’, and as an explanation of these behaviours [64].

Second, several parents expected an ASD diagnosis to provide them with the recognition by others of the *legitimacy* of their worries about the child. It has been argued that all people need to “be able to ‘pass’ as competent, to be ‘normal’. If we falter in our attempts to ‘pass’, it is important that we have a reason or an account of our behaviour that is believable in our community and that provides an acceptable explanation, a reasonable account, of why we cannot pass or do something normally expected” (Edgerton 1967 in ([65], p222). Some scholars have argued that at this moment in the Western ‘neurobiological age’, biomedical and neurological explanations of behaviours are highly valued ‘accounts’ in the sense that they are widely accepted as reasons why a child (or adult) fails to ‘pass’ [66, 67].

Third, the parents regularly predicted that an ASD diagnosis would *reduce expectations* towards them as parents and towards the child, both on behalf of themselves and others. Many parents valued this anticipated implication of an ASD diagnosis but, as described above, at the same time, they also regularly feared the others-toward-child part of it as a possibly stereotyping reaction of other people towards their child. Those parents were afraid that a diagnosis might prompt people to expect less from the child *because he had ASD*. This fear can be linked to the aforementioned study on the perception of incompetence in persons with neuro-cognitive disorders [63]. Instead, several parents said to prefer people to adapt their expectations to the specific ‘profile’ of their child, for example if the child would develop better than expected ‘for a child with ASD’.

Fourth, many parents anticipated that a diagnosis would have an *exculpatory* effect for themselves and the child, both on behalf of them as parents and of others. These parents’ anticipation of such an effect is related to the questions raised by both parents and people in their vicinity regarding parents’ contributory role to the child’s difficult or different behaviours due to the parents’ child-rearing practices. Concerning this anticipated effect towards the child, in a qualitative study on adult experiences of their diagnosis, the ASD diagnosis was termed ‘the not guilty verdict’ [68]. However, neurobiological explanations were found not to be linked to reduced blame in correlational and experimental studies [61].

### Clinical implications

In sum, we found that the parental experiential journey towards an ASD diagnosis was dominated by parents’ expectations of several specific implications if their child would get an ASD diagnosis. Therefore, we argue that communicating about these parental expectations prior to a diagnostic ASD assessment will help clinicians to better understand parents’ requests for help, and to address their needs more effectively. Indeed, in the scientific literature on medical care in general, the active involvement of the patient –parents^1^ when it concerns very young children– by the clinician during the clinical consultation process has been advocated both in the ethical and medical literature. The recommended type of clinician-patient relationship or medical practice goes under various names, like shared decision making, person-centred practice, participatory medicine [71–73], and, specifically with regard to diagnosis, person-centred integrative diagnosis [74]. For example, in shared decision making, it is argued that by recognising parents’ experiences and anticipations, clinicians may be more responsive to parents’ needs and thus become ‘better’ clinicians [71].

### Limitations

This study has several limitations. First, this is a qualitative study conducted in one cultural region, so study results can only be transferred to another region and society with the appropriate translational adaptations [51]. In our experience both with clinical work and international literature however, Belgium can be considered to be a quite typical Western country in regard to the studied topic. The limited sample size is in line with the recommendations of qualitative studies [45] and half of the parents asked to participate declined the invitation. A selection bias cannot be excluded since we purposively sampled parents who asked for a diagnostic ASD assessment of their child. These parents had made up their mind that they wanted such an assessment, so arguably the participants presumed that the balance between the positive and negative implications of a diagnosis would be in favour of the former ones. Also, 9 out of 17 parents had an older child diagnosed with ASD, so their experiences of the diagnosis in the younger child was without any doubt influenced by their experiences with the older child. According to the IPA guidelines, we inductively analysed each interview within the participant’s personal context in order to also discern all effects of this particular difference between the parents. For example, we used separate codes when parents talked about experiences related to the fact that they had an older child diagnosed with ASD. Moreover, we only interviewed parents of boys without a previous diagnosis of disability. Parents’ experiences concerning an ASD diagnosis in their child may differ if the child is a girl or has already been diagnosed with a disability. We chose to exclude children with a previous diagnosis of disability because such a diagnosis might influence or even ‘overshadow’ parents’ experiences of the ASD diagnosis per se. Finally, each research method has inherent limitations, so we used many recommended strategies to enhance the trustworthiness of a qualitative study’s findings. For example, the research team was reflexive throughout the study in order to address potential biases resulting from each researcher’s professional and personal perspectives on the studied topic [51].

## Conclusions

This paper draws an overview of the numerous implications the interviewed parents anticipated when their child would be diagnosed with ASD. Our findings suggest several issues for clinicians to bear in mind during a consultation with parents who request a diagnostic ASD assessment of their young child. While requesting help, parents may have ambiguous feelings about the possible ascription of an ASD diagnosis to their child. They may expect both positive and negative implications of an ASD diagnosis, and look forward not only to treatment-related but also to various psycho-relational implications of a diagnosis. Purposively bringing up and transparently discussing these topics during a clinical consultation will enhance the working alliance and communication between parents and clinician, and will lead to a more satisfying clinical trajectory for parents.

## Supplementary information

**Additional file 1.** COREQ Checklist. Consolidated criteria for Reporting Qualitative research Checklist.

**Additional file 2.** Parents’ experiences prior to their child’s diagnostic assessment. Themes and subthemes resulting from the Interpretative Phenomenological Analysis of the interview data.

## Data Availability

The datasets used and/or analysed during the current study are available from the corresponding author on reasonable request.

## Abbreviations

ASD: Autism spectrum disorder
DSM: Diagnostic and statistical manual of mental disorders
ID: Intellectual disability
IPA: Interpretative Phenomenological Analysis

## Footnotes

1 Since this study focused on very young children, we did not investigate the children’s own experiences but the experiences of their parents, who have been found to be indispensable for children in the ASD process [69, 70].
