# Supplementary material for: Parents’ multi-layered expectations when requesting an Autism Spectrum Disorder assessment of their young child: an in-depth interview study
Source: BMC Psychiatry. 2020 Sep 10;20:440. doi: 10.1186/s12888-020-02806-7 (PMC7488345; doi:10.1186/s12888-020-02806-7)
Supplement: Supplementary file 2 — Additional file 2. Parents’ experiences prior to their child’s diagnostic assessment. Themes and subthemes resulting from the Interpretative Phenomenological Analysis of the interview data. [file 12888_2020_2806_MOESM2_ESM.docx]

Additional file 2

*Parents’ experiences prior to their child’s diagnostic ASD assessment*

| Themes | Subthemes |
| --- | --- |
| Noticing worrisome behaviours in child | Behaviours challenging – not normal   - In comparison to other children - Parents Sib+: also in comparison to older sibling with ASD diagnosis   Questions on child-rearing practices (less in parents Sib+) |
|  | Concerns about parents’ responsibility toward behaviours   - By parents - By others |
| ‘Bidirectional’ involvement of professionals | Parents ask for professionals’ opinion |
|  | Professionals mention their concerns to parents |
| ASD mentioned and diagnostic ASD assessment arranged | Need of early diagnosis – but other people criticizing  Wish for ‘the best’ for child  Tension  Uncertainty about   - What ASD is - Match between ASD and child (gradually less doubts in parents Sib+) - Child’s behaviour part of personality vs. due to ASD   Awaiting conclusive answers from professionals |
| Ambiguous feelings about (consequences of) diagnosis | Anticipated negative implications (already experienced by parents Sib+)   - ASD ‘for life’ (but less ‘fixed’ than ID) - Stigmatising and stereotyping |
|  | Anticipated positive implications (already experienced by parents Sib+)   - Treatment-related: educational approach – entitlement to treatment services - Psycho-relational: understanding – recognition – reduced expectations – exculpation |
|  |  |
|  |  |
